# Positional differences in the wound transcriptome of skin and oral mucosa

**DOI:** 10.1186/1471-2164-11-471

**Published:** 2010-08-12

**Authors:** Lin Chen, Zarema H Arbieva, Shujuan Guo, Phillip T Marucha, Thomas A Mustoe, Luisa A DiPietro

**Affiliations:** 1 Center for Wound Healing & Tissue Regeneration, University of Illinois, Chicago, USA; 2 Core Genomics Laboratory, University of Illinois, Chicago, USA; 3 Department of Surgery, Division of Plastic Surgery, Feinberg School of Medicine, Northwestern University, Chicago, USA

## Abstract

**Background:**

When compared to skin, oral mucosal wounds heal rapidly and with reduced scar formation. Recent studies suggest that intrinsic differences in inflammation, growth factor production, levels of stem cells, and cellular proliferation capacity may underlie the exceptional healing that occurs in oral mucosa. The current study was designed to compare the transcriptomes of oral mucosal and skin wounds in order to identify critical differences in the healing response at these two sites using an unbiased approach.

**Results:**

Using microarray analysis, we explored the differences in gene expression in skin and oral mucosal wound healing in a murine model of paired equivalent sized wounds. Samples were examined from days 0 to 10 and spanned all stages of the wound healing process. Using unwounded matched tissue as a control, filtering identified 1,479 probe sets in skin wounds yet only 502 probe sets in mucosal wounds that were significantly differentially expressed over time. Clusters of genes that showed similar patterns of expression were also identified in each wound type. Analysis of functionally related gene expression demonstrated dramatically different reactions to injury between skin and mucosal wounds. To explore whether site-specific differences might be derived from intrinsic differences in cellular responses at each site, we compared the response of isolated epithelial cells from skin and oral mucosa to a defined in vitro stimulus. When cytokine levels were measured, epithelial cells from skin produced significantly higher amounts of proinflammatory cytokines than cells from oral mucosa.

**Conclusions:**

The results provide the first detailed molecular profile of the site-specific differences in the genetic response to injury in mucosa and skin, and suggest the divergent reactions to injury may derive from intrinsic differences in the cellular responses at each site.

## Background

Wound healing is a complicated pathophysiological process orchestrated by a variety of known and unknown factors. Although cutaneous and mucosal wound healing proceed through the same stages of hemostasis, inflammation, proliferation, and remodeling, mucosal wounds demonstrate accelerated healing compared to cutaneous wounds [1-4]. Mucosal wounds also generally heal with minimal scar formation, and hypertrophic scars are rare in the oral cavity [5].

Studies in at least three different models of oral mucosal wound healing now support the concept that rapid wound closure and reduced scar formation are near-universal features of the superior healing phenotype that is observed in the oral cavity [2,5-7]. The one exception that has been seen is excisional wounds placed on the hard palate of the mouse. In this model, the underlying connective tissue is extremely thin, so the wound depth reaches the periosteal bony surface and healing is slow [8]. Nearly all other oral mucosal wounds, including palatal wounds in humans and pigs, heal more quickly than skin [5,6].

While anatomical differences in mucosal and skin repair have been described, the molecular basis of the privileged repair of mucosal wounds is less well understood. One well-described difference between oral mucosal and dermal healing is the relative decrease in inflammation that is seen in oral mucosal wounds. Oral mucosal wounds contain less infiltrating inflammatory cells [2,6], and lower levels of inflammatory cytokines such as IL-1α, IL-1β, TNF-α, and chemokines such as KC [2,9] (Table 1). In addition, the ratio of TGF-b1/TGF-b3, a factor suggested to predict scar formation, is decreased in oral mucosal wounds [7]. Interestingly, the pattern of angiogenesis is also different in oral mucosal and skin wounds, as the angiogenic response in oral wounds is more highly regulated [1].

**Table 1:** Characteristics of skin and mucosal wounds [1,2,7,9]

	Skin	Tongue
Re-epithelialization at 24 h	40%	100%
Inflammatory infiltrates		
MPO (U/mg protein) at 24 h	2.82 ± 0.2	1.26 ± 0.2
Mϕ (HPF) at 72h	10.8 ± 0.3	6.7 ± 0.4
T cells (HPF) at day 7	15.1 ± 1.5	5.6 ± 0.3
Cytokine/chemokine		
IL-6	↑↑	↑
IL-a	↑↑	↑
IL-1β	↑↑	↑
TNF-α	↑↑	↑
TGF-β1	↑↑	↑
TGF-β3	↑	↑↑
KC	↑↑	↑
Angiogenesis		
Vessel density compared to normal tissue at day 5 (fold increase)	11.5	3.4
VEGF	↑↑	No marked change
Collagen fibril diameter in wound	Decreased	No marked change

Several previous studies have examined the transcriptome in wounds in a more limited fashion than the current study. Microarray analysis has been used to determine the changes in the transcriptome at the edges of acute wounds in murine skin [10], in laser captured blood vessels from human chronic wounds [11], and in non-healing human venous ulcers [12]. Other studies have compared gene expression in wounds produced at early and later gestational ages [13] and in prenatal and postnatal wounds [14]. These studies have contributed to our understanding of the wound transcriptome and the complexity of the repair process. To date, a global and comprehensive profiling of the differentially expressed genes in normal cutaneous and mucosal wounds has not been reported. The purpose of this study was to utilize microarray analysis to discover differences in the repair processes of oral and cutaneous wounds. A systematic profiling of gene expression in matched, equivalent sized cutaneous and oral mucosal wounds was performed at seven time points from 6 hours to 10 days post wounding. Microarray analysis of gene expression in normal, unwounded tissues was also performed. Our results suggest that tissue repair in oral mucosal wounds involves a more rapid, yet more refined response than that of skin wounds. The global expression patterns show that a significantly fewer number of gene sets change over time in mucosal wounds than in skin wounds. These findings demonstrate that tissue repair has distinguishable and different genomic expression patterns in mucosal and cutaneous sites.

## Results

### More gene sets are differentially expressed in the skin wounds than in mucosal wounds over time

**Figure 1:**
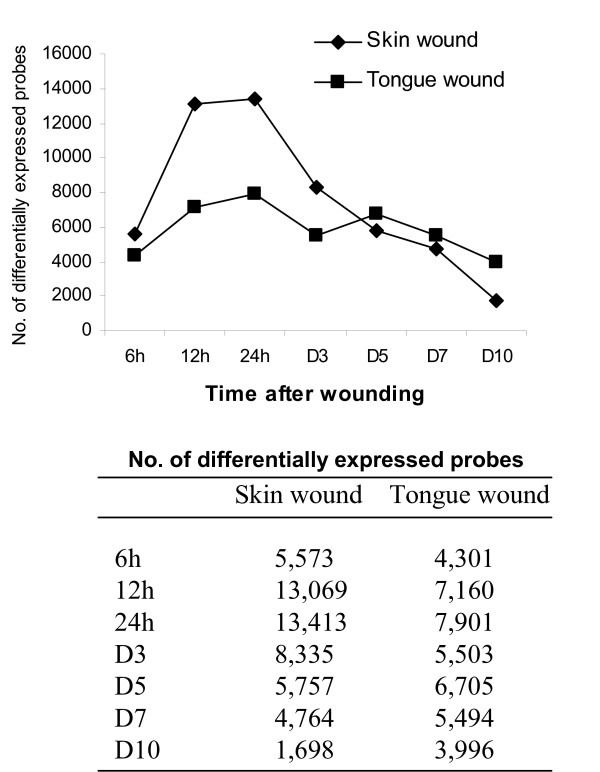
**More genes are significantly expressed in skin wounds than in the tongue wounds**. One mm punch wounds were made on BALB/c mice skin and tongue. Wounds and surrounding tissues were harvested at 6 h, 12 h, and days 1, 3, 5, 7, and 10. Total RNA was extracted. Microarray was carried out using Affymetrix GeneChip arrays. A one-way ANOVA was used to identify significantly differentially expressed gene sets across all time points after wounding (FDR < 0.05). Figure shows the numbers of significantly expressed probes at each time point compared to normal tissues.

As described in detail in Materials and methods section, normalized and background corrected expression values across all time points for each tissue were subjected to one-way ANOVA test. Raw p-values were corrected for False Discovery Rate (FDR) and FDR less than 0.05 was considered as a threshold of significance of differential expression. Using baseline, uninjured matched skin as a control, the results of the test for skin samples report an overall number of significant differentially expressed probe sets as 22,522 out of 45,037 examined. Distribution of significant expressions across time points compared to unwounded skin is depicted on Fig. 1. The numbers indicate that in skin a sharp spike in transcriptional changes occurs at 12 and 24 hours with 13,069 and 13,413 of gene sets respectively after wounding. The numbers quickly and significantly subside from day 3 thereafter. For the tongue wound, again using unwounded matched tissue as a control, the overall number of significant differentially expressed probe sets was 19,817, which is slightly less than the overall number of significantly expressed gene sets for skin. The distribution of significant differential expression across the time points (Fig. 1) shows that transcriptional changes in the tongue occur at relatively even rates across all time points. Skin and mucosal wounds undergo a comparable degree of transcriptional changes except at 12 and 24 hours after injury. Skin wounds are significantly more reactive than tongue wounds at 12 and 24 hours after injury. This time period accounts for 85% of the overall changes in skin and 95% of the difference between skin and tongue based on the number of probe set IDs.

### Differentially expressed clusters of gene sets identified in skin and tongue wounds

**Figure 2:**
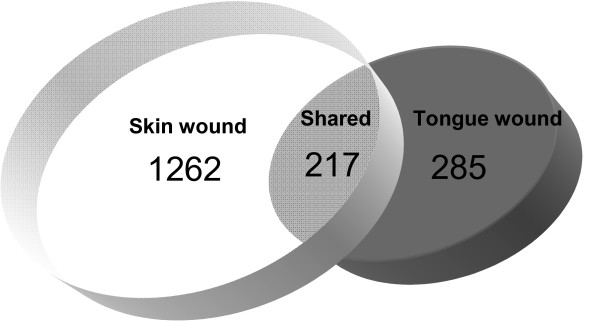
**Numbers of tissue specific and commonly expressed probes in the skin and tongue wounds**.

To obtain differentially expressed clusters of gene sets in skin and tongue wounds, an additional filtering was performed in gene sets identified in One-way ANOVA test only using those with FDR corrected p value < 1E-5 and absolute fold change ≥ 2 in at least one time point. Filtering produced a list of 1,479 probe sets for skin and 502 probe sets for tongue significantly differentially expressed over time (Table 2), suggesting that the response to wounding in tongue is more refined than that of skin. Before K-means clustering, log2 intensity values were normalized by standard deviation to correct for differences in the scale of expression intensity. Of the 1,479 probe sets that were differentially expressed over the course of repair in skin, 1,262 (85%) were expressed only in skin wounds and not in tongue. Of 502 gene sets found to be differentially expressed in tongue wounds, 285 (56%) were specific to tongue wounds and not found in skin wounds. 217 probe sets were found to be common between the two wound types (Fig. 2).

**Table 2:** Patterns of K-means cluster distribution

Skin				
Cluster	Members (#)	Skin only (#)	Designation	Pattern (%)
1	95	68	Early up-regulation (high)*	
2	264	219	Early up-regulation (medium)*	64.0 (cluster 1, 2, & 3 combined)
3	588	521	Early up-regulation (low)*	
4	331	295	Early down-regulation	22.4
5	201	159	Late up-regulation	13.6
Total	1,479	1,262		
				
**Tongue**				
**Cluster**	**Members (#)**	**Tongue only (#)**	**Designation**	**Pattern (%)**
1	27	7	Early up-regulation (high)*	
2	112	66	Early up-regulation (medium)*	66.5 (cluster 1, 2, & 3 combined)
3	195	121	Early up-regulation (low)*	
4	52	24	Early down-regulation	10.4
5	116	67	Late up-regulation	23.1
Total	502	285		

Table contains information on distribution of Affymetix probe sets between k-means clusters in skin and tongue.

**Cluster**: k-means cluster assignment.

**Members**: number of Affymetrix probe sets within the cluster.

**Skin/tongue only (#)**: number of tissue specific probe sets.

**Skin/tongue only (%)**: percentage of tissue specific probe sets.

**Designation (level of change)**: name assigned to a group with indication of the amplitude of differential expression.

**Pattern (%): **percentage of the numbers of probe sets in each pattern against total numbers of probe sets.

*indicates clusters combined for later functional analysis

**Figure 3:**
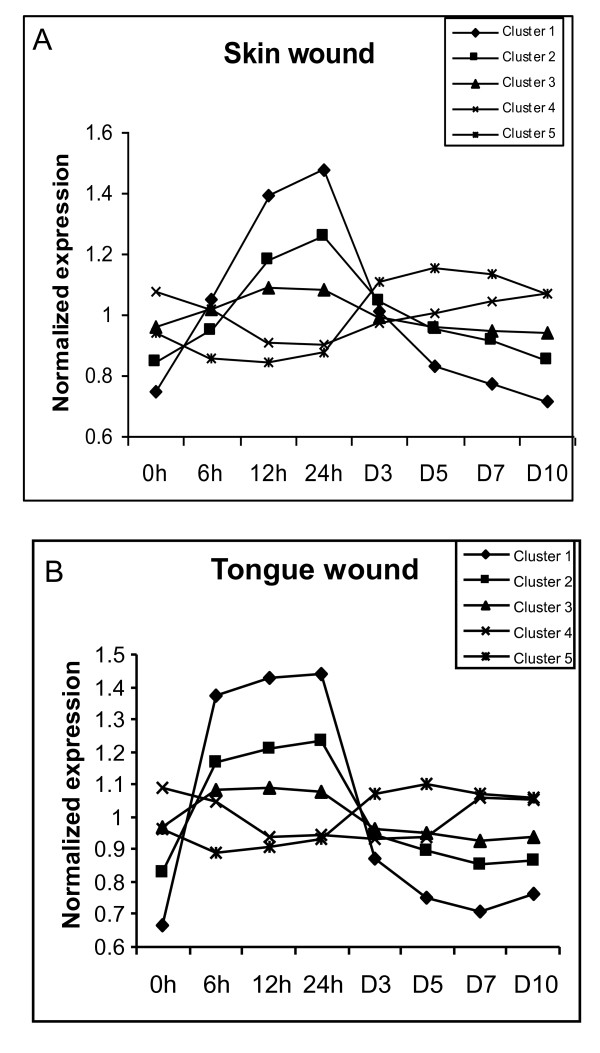
**Clusters of differentially expressed probe sets in skin and tongue wounds**. K-means clustering of filtered transcripts revealed 5 clusters of behaviors of differentially expressed probe sets in each type of wound that demonstrated similar expression patterns over the course of healing.

**Figure 4:**
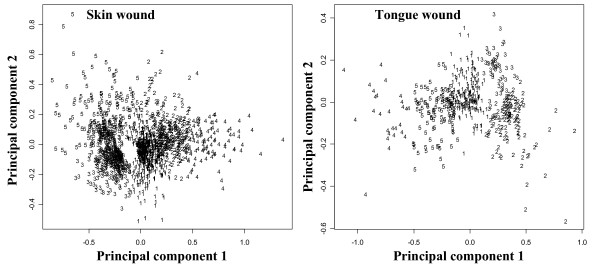
**Results of k-means clustering projected into the plain of main principal component**.

The results of k-means clustering are shown on Fig. 3 and Table 2, where cluster centers are plotted against time points of sample collection. A number of clusters for k-means clustering analysis were chosen equal to five; validation of the number of clusters was obtained by principal component analysis (Fig. 4).

K-means cluster analysis identified a few patterns of transcriptional changes in healing tissues (Fig. 3). In skin, the largest group of transcripts (k-means clusters 1, 2, and 3 comprised of 95, 264, and 588 gene sets respectively) undergoes rapid early up-regulation, plateaus at 12-24 hours and rapidly subsides by day 3 and more gradually thereafter. The difference between the three clusters lays mainly in the degree of up-regulation (from low to medium to high). The whole group represented by these three clusters is designated as early upregulated (Fig. 3A and Table 2). Another group of transcripts (k-means cluster 4 comprised of 331 gene sets) undergoes early down-regulation, reaches its lowest point at 12-24 hours and gradually returns to the initial expression level by day 10. This group was designated as skin early downregulated (Fig. 3A and Table 2). The expression of the last group of transcripts (k-means cluster 5 comprised of 201 gene sets) slightly decreases during the first 24 hours and then undergoes a low degree of up-regulation that peaks at day 3 and remains upregulated up to day 10. This group was designated as late upregulated (Fig. 3A and Table 2).

In tongue wounds, the largest group of transcripts (k-means clusters 1, 2, and 3 comprised of 27, 112, and 195 gene sets respectively) undergoes rapid early up-regulation, plateaus at 6-24 hours and subsides sharply by day 3 and gradually decreases thereafter. Similar to skin wounds, the difference between the three groups also lays mainly in the degree of up-regulation (from low to medium to high). The difference between skin and tongue wounds is that in tongue wounds up-regulation reaches its height at an earlier time point (6 hours in tongue vs. 12 hours in skin). This group, represented by three k-means clusters, was designated as early upregulated (Fig.3B and Table 2). Another group of transcripts (k-means cluster 4 comprised of 52 gene sets) is down regulated at 6 hours, and reaches its lowest point between 12 hours to day 5, then returns to initial expression level by day 7 after wounding. This group remains downregulated longer than the analogous group in skin wounds. This group was designated as early downregulated (Fig. 3B and Table 2). The last group of transcripts (k-means cluster 5 comprised of 116 gene sets) resembles the analogous group in skin wounds (k-means cluster 5) and is slightly down regulated in the first 24 hours after wounding, then upregulates at day 3 and remains elevated up to day 10. This group was designated as late upregulated (Fig. 3B and Table 2).

As described above, tongue wounds contained expression groups that were highly similar in pattern to those identified in skin. While the overall number of genes differed, the percentage of gene sets found in each expression group was quite similar in the two tissues (Table 2). In skin, 64% of all identified transcripts fell into the early up-regulated transcripts group, while 66.5% of the identified transcripts in tongue wounds were situated within the early up-regulated group. In addition, there were 22.4% and 13.6% of gene sets in the skin wounds, and 10.4% and 23.1% of gene sets in the tongue wounds that fell into the early down-regulated and late up-regulated transcripts group respectively (Table 2).

### Functional motifs behind transcriptional changes

To uncover cellular processes likely affected by transcriptional changes, we applied gene enrichment and functional annotation analysis with the use of the web based application DAVID (Database for Annotation, Visualization and Integrated Discovery) at http://david.abcc.ncifcrf.gov.

Functional annotation and gene functional classification analysis were performed. Functional annotation tool provides batch annotation and highlights biological terms associated with a list of gene sets by means of calculating significance of the enrichment of known functional groups, such as gene ontology (GO) categories or Kyoto Encyclopedia of Genes and Genomes (KEGG) pathways, with experimentally derived differentially expressed genes. A list of top ranking functional terms with corresponding statistical parameters are given in Table 2a, 3a, and 4a which correspond to early upregulated genes, early downregulated genes, and early down/late upregulated genes respectively. Functional gene classification analysis generates gene-to-gene similarity matrix based on functional annotations associated with each gene and classifies genes into functionally related groups. Outputs display lists of enriched consensus terms associated with each group. By cross-referencing these two types of outputs, we were able to address similarities and differences between the two tissues. The following results were obtained:

**Table 3:** Early up-regulated genes: enriched annotation terms

	Skin		Tongue	
Terms	Members (#)	p value	Members (#)	p value
GO: Response to wounding	52	3.3E-16	25	5.8E-9
GO: Inflammatory response	43	1.2E-15	24	5.7E-11
SP_PIR_KEYWORDS: Cytokine	39	1.0E-15	16	6.1E-6
KEGG: Toll-like receptor signaling pathway	31	4.5E-12		
GO: Receptor binding	73	1.2E-11	28	3.6E-4
KEGG: Cytokine-cytokine receptor interaction	47	1.2E-11	18	9.1E-4
KEGG: Jak-STAT signaling pathway	29	1.8E-6		
GO: Chemotaxis	25	4.0E-10	11	6.4E-4

Table contains a list of top significant annotation terms and p values derived from DAVID functional annotation analysis.

**Terms**: GO, SP_PIR_KEYWORDS or KEGG.

**Member (#)**: numbers of differentially expressed transcripts associated with the term.

P **value**: modified Fisher Exact p-value, FDR adjusted.

**Table 4:** Early up-regulated genes: enriched groups of functionally related genes

	Skin		Tongue	
Terms	Members (#)	Score	Members (#)	Score
Interferon α, β and δ/cytokine	16 (Group 1)	9.77		
Chemokine	12 (Group 2)	9.58	10 (Group 1)	6.46
Cell differentiation/apoptosis	8 (Group 3)	3.85	1	
Positive regulation of protein kinase activity	4 (Group 4)	3.68		
Regulation of metabolic/biological process	4 (Group 5)	2.91		
Pattern recognition receptors (TLR related)	7 (Group 6)	2.82		
Ribosome/organelle organization	8 (Group 7)	2.5		
Matrix metallopeptidase (MMP)	6 (Group 8)	2.04		
Negative regulation of cell proliferation			4 (Group 2)	3.34
GTP binding			14 (Group 3)	3.08
Keratinization/epidermal development			4 (Group 4)	2.27

Table contains a list of top enriched groups of annotation terms and enrichment scores derived from DAVID gene functional classification analysis.

**Term**: consensus annotation terms shared by a group of genes.

**Member (#)**: numbers of differentially expressed transcripts associated with the term.

**Score**: statistical significance of the enrichment based on overall EASE scores.

#### Functional Annotation of Early Upregulated Genes

We found that both types of wounds exhibited similar biological tendencies in early response (clusters 1, 2, and 3 from K means analysis) including those identified as 1) response to wounding, 2) inflammatory response, 3) cytokine, 4) cytokine-cytokine receptor interaction and 5) chemotaxis (Table 3). However, a number of group members is higher in skin, therefore the majority of adjusted p values for skin wounds were lower than that for tongue wounds (Table 3). In addition, analysis identified the cytokine-cytokine receptor interaction pathway as significantly expressed in both skin wounds and tongue wounds (Table 3 and additional file 1) with more numbers of cytokine/chemokines observed in skin wounds. Interestingly, the Toll-like receptor-signaling pathway (Table 3 and additional file 2) and Janus family tyrosine kinases-signal transducers and activators of transcription (JAK-STAT) signaling pathway (Table 3 and additional file 3) were only utilized in skin wounds. Furthermore, the DAVID gene functional classification analysis identified 27 groups in skin wounds but only 11 groups in tongue wounds. When 2.00 was used as the cutoff for enrichment scores, the number of groups found to be significantly enriched was reduced to 8 out of 27 in skin wounds (Table 4 and additional file 4) and just 4 out of 11 in tongue wounds (Table 4 and additional file 5).

The group of genes (cytokines) with the highest enrichment score (9.77) in skin wounds included interferon (IFN)-α, IFN-β, IL-23, IL-24, and CSF-3. None of these genes were found to be differentially expressed in tongue wounds (Additional file 4 and 5).

Chemokines were found to be prominently differentially expressed by wounds at both locations. Functional grouping gene demonstrated that Group 1 in tongue wounds and Group 2 in skin wounds had 10 and 12 chemokines respectively. Both wound types included CCL2, CCL4, CCL7, CXCL1, CXCL2, CXCL5, and CXCL11. However, CCL5, CCL12, and CXCL10 only appeared in tongue wounds, while CCL3, CCL20, CXCL3, CXCL7, and CXCL13 only appeared in skin wounds (Additional file 4 and 5).

One interesting set of genes that was found to be enriched in mucosa, but not in skin, was a group of several genes involved in the negative regulation of proliferation. More specifically, Group 2 in tongue wounds contained 4 such genes including IFN induced transmembrane protein 3, schlafen 1, schlafen 2, and schlafen 3 (Additional file 5). This group of genes was not identified as enriched as a group in skin wounds, although each was significantly expressed in skin wounds according to ANOVA.

Group 3 genes in skin wounds included myeloid cell leukemia sequence 1, pleckstrin homology-like domain, family a, member 1, lectin, galactose binding, soluble 7, egl nine homolog 3, B-cell leukemia/lymphoma 2 related protein a1a, B-cell leukemia/lymphoma 2 related protein a1 d, B-cell leukemia/lymphoma 2 related protein a1b, and axin1 up-regulated 1 in skin wounds are associated with cell differentiation and apoptosis (Additional file 4). Only one of these genes (lectin, galactose binding, soluble 7) was present in tongue wounds.

Group 3 in tongue wounds contains 14 gene members associated with GTP-binding activity (Additional file 5).

Group 4 in tongue wounds included small proline-rich proteins 2d, 2f, 2i, 2h and 2j which are associated with keratinization and epidermal development (Additional file 5). Small proline-rich protein 2a, 2d, and 2i were present in skin wounds, but not enriched as a group.

Group 4 in skin wounds contains a groups of 4 genes which are involved in positive regulation of protein kinase activity, including growth arrest and DNA-damage-inducible 45 β, sperm associated antigen 9, toll-interleukin 1 receptor (tir) domain-containing adaptor protein and arginine vasopressin-induced 1 (Additional file 4). None of these genes appeared as differentially expressed in tongue wounds.

Group 5 in skin wounds includes hematopoietic cell specific lyn substrate 1, cardiotrophin-like cytokine factor 1, CD80 antigen and yamaguchi sarcoma viral (v-yes-1) oncogene homolog. All of these genes play a positive role in regulation of metabolic/biological process (Additional file 4). None of these genes were present as differentially expressed in tongue wound.

Group 6 in skin wounds includes toll-like receptor (TLR) 2, TLR 4, TLR 6, TLR 13, c-type lectin domain family 7, member a, MyD88, and IL-1b. These molecules, with the exception of MyD88 and IL-1, are pattern recognition receptors for pathogens and/or endogenous ligands and play critical roles in innate immunity (Additional file 4). None of these genes appears as differentially expressed in tongue wound.

Group 7 in skin wounds includes ebna1 binding protein 2, bystin-like, tsr1/20 s rRNA accumulation/homolog (yeast), riken cDNA 4933411h20 gene, sda1 domain containing 1, nuclear import 7 homolog (S. cerevisiae), riken cDNA 5730405k23 gene, and riken cDNA 2610012o22 gene. These genes are all intracellular membrane-bound organelle and nucleus proteins that play roles in ribosome and organelle organization, biogenesis, and assembly (Additional file 4). None of these genes appear as differentially expressed in tongue wound.

Group 8 in skin wounds includes matrix metallopeptidase (MMP) 1a, MMP 1b, MMP 8t MMP 9, MMP 10, and MMP 13 (Additional file 4). None of these genes appears as differentially expressed in tongue wound.

#### Functional Annotation of Early Downregulated Genes

The early down-regulated genes included those identified in Cluster 4 (K means analysis). Functional annotation clustering analysis of the early down-regulated genes in both wounds identified two major overlapping functional terms: DNA dependent transcription and DNA binding (Table 5). However, skin wounds had higher enrichment scores and lower p values than those of tongue wounds because of the larger number of members (Table 5).

**Table 5:** Early down-regulated genes: enriched annotation terms

	Skin		Tongue	
Terms	Members (#)	p value	Members (#)	p value
GO:Transcription, DNA-dependent	49	4.0E-4	9	2.3E-1
GO: DNA binding	47	6.9E-4	5	5.0E-1

Table contains a list of top significant annotation terms and p values derived from DAVID functional annotation analysis.

**Terms**: GO

**Member (#)**: numbers of differentially expressed transcripts associated with the term.

**P value**: modified Fisher Exact p-value, FDR adjusted.

Gene functional classification analysis of genes from skin identified a group of 52 genes with an enrichment score of 3.0 (Table 6 and additional file 6). This group included 45 genes associated with transcription regulation, including 38 DNA binding proteins and 11 members of transcription factor complexes. The group included both positive and negative regulators.

**Table 6:** Early down-regulated genes: enriched groups of functionally related genes

	Skin		Tongue	
Terms	Members (#)	Score	Members (#)	Score
Transcription regulation/DNA binding	52 (Group 1)	3.0	5 (Group 1)	1.25

Table contains the top enriched group of annotation term and enrichment scores derived from DAVID gene functional classification analysis.

**Term**: consensus annotation terms shared by a group of genes.

**Member (#)**: numbers of differentially expressed transcripts associated with the term.

**Score**: statistical significance of the enrichment based on overall EASE scores.

Gene functional annotation clustering analysis of Cluster 4 genes suggests that the tongue wounds have similar gene clusters as the skin wounds but has much less numbers of members and higher p-values (Table 5). Only one functional group was identified in gene functional classification analysis; this group had an enrichment score of 1.25 and contained 5 transcripts annotated as regulators of transcription, and 4 as DNA binding (Table 6 and additional file 7). When the results of the functional classification analysis from skin and tongue were compared, four members of this group in tongue wounds except nuclear receptor subfamily 1, group d, member 1 are present in skin as well.

#### Functional Annotation of Late Upregulated Genes

Cluster 5 derived from K-means analysis represents those genes showing a pattern of late upregulation. DAVID functional annotation clustering analysis showed significantly enriched annotation terms that are shared by both tissues include extracellular matrix (ECM), collagens, structural proteins, ECM-receptor interaction, cell communication, and peptidase activity (Table 7). While the terms that are identified are the same, all skin terms contain more gene members and have higher enrichment scores and lower adjusted p values (Table 7). Several ECM proteins seem to be skin specific, such as osteomodulin, leprecan 1, biglycan 1, a disintegrin-like, and metallopetidase (reprolysin type) with thrombospondin type 1 motif 16, nidogen 2, fibrillin 2, and microfibrillar-associated protein 2. More detailed analysis based on DAVID gene functional classification analysis reveals that in both types of wounds the most significant changes occur within a group of transcripts that encode structural components of procollagens (Table 8, group 1 in additional file 8 and 9).

**Table 7:** Late up-regulated genes: enriched annotation terms

	Skin		Tongue	
Terms	Members (#)	p value	Members (#)	p value
SP_PIR_KEYWORDS: Extracellular matrix	21	5.2E-15	12	1.8E-6
SP_PIR_KEYWORDS: Collagen	14	1.5E-11	9	2.9E-6
SP_PIR_KEYWORDS: Structural protein	13	4.9E-9	10	1.5E-6
GO: ECM-receptor interaction	10	6.5E-6	9	1.4E-5
KEGG: Cell communication	9	1.6E-3	8	1.7E-3
KEGG: Peptidase activity	12	9.5E-1	4	1.0E-1

Table contains a list of top significant annotation terms and p values derived from DAVID functional annotation analysis.

**Terms**: SP_PIR_KEYWORDS, GO, or KEGG.

**Member (#)**: numbers of differentially expressed transcripts associated with the term.

**P value**: modified Fisher Exact p-value, FDR adjusted.

**Table 8:** Late up-regulated genes: enriched groups of functionally related genes

	Skin		Tongue	
Terms	Members (#)	Score	Members (#)	Score
Collagen	13 (Group 1)	9.61	9 (Group 1)	6.58
Serine-type endopeptidase activity	4 (Group 2)	3.05		
EGF-like calcium-binding	4 (Group 3)	2.83	2	
Metalloendopeptidase activity	3 (Group 4)	2.22		
Cytoskeleton	8 (Group 5)	2.08	5 (Group 2)	3.57

Table contains a list of top enriched groups of annotation terms and enrichment scores derived from DAVID gene functional classification analysis.

**Term**: consensus annotation terms shared by a group of genes.

**Member (#)**: numbers of differentially expressed transcripts associated with the term.

**Score**: statistical significance of the enrichment based on overall EASE scores.

Another functional group found in both types of wounds contains transcripts encoding elements of cytoskeleton (group 2 in tongue and group 5 in skin) (Table 8, additional file 8 and 9). These functional groups include actin, myosin, and troponin (Additional file 8 and 9). Skin wounds contain cardiac actin α, cardiac troponin t2, myosin/heavy polypeptide 3/skeletal muscle/embryonic, troponin i/skeletal/slow 1, troponin c/cardiac/slow skeletal, myosin/heavy polypeptide 13/skeletal muscle, kinesin family member 20a, and cDNA sequence bc056349. Similarly, tongue wounds were found to contain all of these genes except for the last three.

In skin wounds, functional Group 2 contains 4 peptidases including protease serine 19 (neuropsin), tryptase α/β1, transmembrane protease serine 11a, and protease serine 35 (Additional file 8). None of the members are present in tongue wounds. Group 4 in skin wounds also contained peptidases, including matrix metallopeptidase 23, a disintegrin/metallopeptidase domain 12, and a disintegrin-like and metallopeptidase with thrombospondin type 1 motif, 16 (Additional file 8). None of these genes were present in tongue wounds.

Functional Group 3 in the skin contains 4 calcium binding proteins including hypothetical protein 9430004m15, CD248, cadherin 11, and thrombospondin 4 (Additional file 8). CD248 and thrombospondin 4 are also involved in adhesion. Tongue wounds contained only the first two genes in this group.

### Skin and mucosal keratinocytes respond differently upon IL-1β stimulation

**Figure 5:**
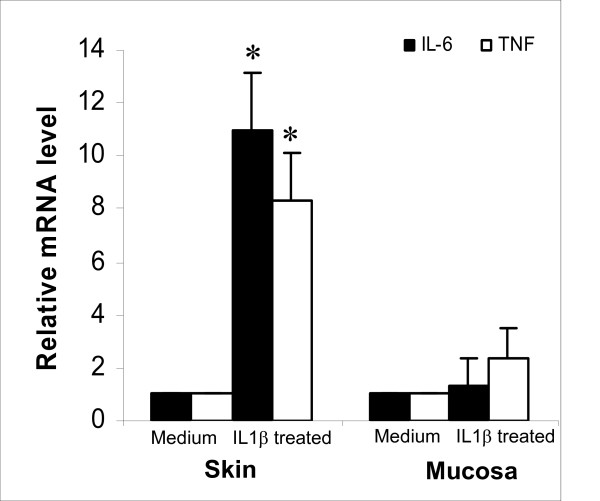
**Differential response of skin and mucosal keratinocytes to IL-1β treatment**. Primary skin and oral mucosal keratinocytes isolated from adult human were treated with recombinant human IL-1β for 24 hours. Relative mRNA expression (fold change) of IL-6 and TNF-α compared to medium treated cells was determined by real time PCR. * p < 0.01 between skin and mucosal keratinocytes for IL-6 and TNF-α (n = 3).

The above microarray identified a number of major differences in the response to injury between skin and mucosa. For example, skin wounds exhibited dramatically more genes related to the inflammatory response than mucosal wounds. To further explore the possibility that this divergence is due to intrinsic differences in the genetic responses of the resident cells of the two tissues, we examined the responses of isolated epithelial cells from skin and mucosa to a defined in vitro stimulus. Individual primary cultures of human epithelial cells from skin and oral mucosa were stimulated with IL-1β, a major inflammatory cytokine that is increased in the wounded tissues. Following stimulation, the amount of IL-6 and TNF-α mRNA that was produced by cells from skin and oral mucosa was compared using real time PCR. Whereas epithelial cells from skin exhibited an 11.0 and 8.3 fold increase in mRNA expression of IL-6 and TNF-α respectively, mucosal epithelial cells showed only a 1.3 and 2.4 fold increase of IL-6 and TNF-α mRNA respectively (Fig. 5). This data supports the concept that the large differences seen in the genomic response to injury in skin and mucosa are derived of at least in part from intrinsic differences in the genetic regulation of cells at each site.

#### Validation of microarray Data

Using gene specific one step RT-PCR and Glucuronidase β and β-actin as a normalization control, relative amounts of CCL27, CSF1, IL-1β, Collagen Ia2, and Collagen III a1 mRNA were determined. CSF1 and IL-1β were found to be significantly increased at 24 h after wounding in both tongue and skin, but not at day 10 after wounding (Table 9), which corresponds to microarray data that identifies these two genes as belonging to an early upregulated cluster (cluster 1, 2 and 3). CCL27 was found to be significantly decreased in the 24 h wounded skin sample only (Table 9), which matches microarray data that this gene belongs to an early downregulated cluster (cluster 4). Collagen Ia2 and Collagen III a1 were found to be slightly decreased at 12 h and significantly increased at day 10 after wounding in both skin and tongue (Table 9). These results correspond to the microarray data showing that these two genes are in an early down and late upregulated cluster (cluster 5). Similar results were obtained when using β-actin as the normalization control (data not shown). CCL2 and CXCL2 mRNAs could be detected at 24 h only, but not in unwounded and day 10 wounded samples in both tongue and skin tissues (data not shown), which in general matches the microarray data. Therefore, RT-PCR confirmed the differential expression patterns of these selected genes.

**Table 9:** Real time RT-PCR validation (Ratio of target/Glucuronidase β)

	Skin		Tongue	
	12 h or 24h	Day 10	12 h or 24h	Day 10
CCL27	-5.45164 (24h)	-1.73222	-1.18748 (24h)	3.67
CSF1	3.368982 (24h)	-1.40295	1.811163 (24h)	1.03867
IL-1β	4598.684(24h)	6863.654	384.8839 (24h)	126.2173
COL Ia2	-1.57193 (12h)	1.825174	-1.08959 (12h)	1.662603
COL IIIa1	-1.58234 (12h)	3.512393	1.104019 (12h)	4.839327

## Discussion

This is the first systemic, comprehensive and dynamic study of gene expression profiles in skin and mucosal wounds over all stages of wound healing. Using well established mouse models of skin and mucosal wound healing [1,2,7] and advanced microarray technology, similarly expressed as well as significantly differentially expressed genes in skin and mucosal wounds were successfully identified. Overall, the identification of five clusters of genes showing similar patterns of expression allowed for a general comparison of the global genomic response to injury in skin and tongue. This comparison suggests that the patterns of expression are similar in both types of wounds, but hardly identical. The results clearly show that the comprehensive genomic response to injury in the tongue is more rapid, shorter in duration, and of lesser intensity than the response of skin to a similar sized insult. This data implies that, as compared to skin, the tongue has an intrinsic genetic response that accelerates repair. One possible reason for the apparent increase in the intensity of the response to injury in skin as compared to tongue could be that baseline expression levels are simply higher in tongue for many genes. In this case, the mucosa, being "preactivated" would not require an increase in the expression of genes during the healing process. To examine this, we compared the baseline levels of several genes that are highly upregulated in the early skin wounds but not tongue wounds. Because inflammation is quite different at the two sites, we focused on genes from the cytokines/chemokines group, including IFN-α, IFN-β, IL-23, IL-24, CSF-3, CCL3, CCL20, CXCL3, CXCL7, and CXCL13. This analysis showed that the baseline levels of nine of these genes are very similar in normal mucosa and skin. The one exception was CXCL13, which is indeed higher at baseline in mucosal sites (Additional file 10). Therefore, any baseline differences can only partially explain the differential healing responses of skin and mucosa.

**Figure 6:**
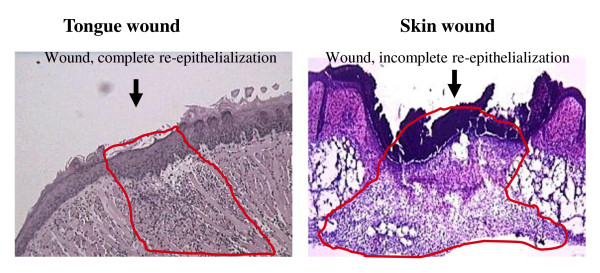
**Wound closure is more rapid in oral wounds**. Pictures shown are representatives of H&E stained 1 mm skin and tongue wounds 24 hours after wounding. Wounded areas are circled.

**Figure 7:**
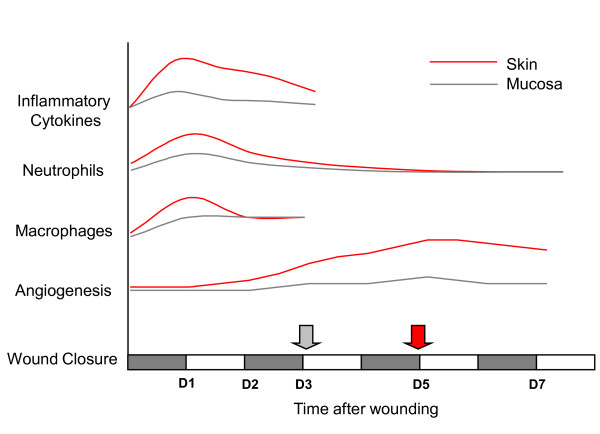
**Time line of specific aspects of wound healing in mucosa and skin**. The relative timing and abundance of inflammatory cytokines, neutrophils, macrophages, and angiogenesis is shown for mucosal and skin wounds, as well as the time of wound closure (arrows). Mucosal wounds close much more rapidly than skin; all other aspects of repair are temporally similar at the two sites. Overall, the magnitude of the response is much less in mucosa, suggesting that mucosa heals by a simpler restoration process than the inflammatory repair process observed in adult skin. Adapted from references [1,2].

In the present study, the gene expression profiles of skin and tongue wounds were compared at the same chronologic time points after injury. An argument might be made that a better comparison would be to compare across those time points that correspond to the same stages of repair, such as the time of wound closure or the time of maximum inflammation. The decision to use direct, identically timed samples from each site was made after reviewing the healing patterns of oral and skin wounds. Our previous studies [1,2,7] in this model clearly show that mucosal wounds close much more rapidly than skin (Fig. 6 and reference [7]. However, all other aspects of repair seem temporally similar at the two sites. For example, while the magnitude differs, the peak abundance of inflammatory cytokines, neutrophils, and macrophages occurs at nearly the same time in wounds from both anatomic locations (Fig. 7). Similarly, although the angiogenic response is decreased in mucosal wounds, the time point of maximum vascularity is the same in both sites. With the exception of wound closure, the timing of the response to injury is similar in both tissues; this finding indicates that time-matched samples should be informative. Importantly, though, the magnitude of the response is much less in mucosa, suggesting that mucosa heals by a simpler restoration process than the one utilized by adult skin.

The comprehensive analysis of the transcriptome of skin and mucosal wounds allowed us to identify several specific groups of genes that are disparately expressed in the skin and mucosal wounds and that may be functionally important to healing outcomes. Skin and mucosal wounds demonstrated notable differences in the expression of proinflammatory and inflammation related elements. Mucosal wounds exhibited decreased levels of pro-inflammatory genes including cytokines and chemokines. Cytokine and chemokine induced inflammation in the early stage of wound healing is critical for tissue reconstruction and regeneration in the later stages, as several studies show that an appropriate inflammatory response in the early stage is critical for wound healing [15-18]. Cytokines and chemokines play important roles in the inflammatory response including recruiting and activating inflammatory cells such as neutrophils, macrophages, T cells, and mast cells, which are all involved in the wound healing process [15-18]. Among the early response genes, cytokines including IL-23, IL-24, CSF, IFN-α, and IFN-β and chemokines including CCL3, CCL20, CXCL3, CXCL7, and CXCL13 are significantly expressed in skin wounds but not in mucosal wounds. This data suggests that decreased inflammatory cytokine and/or chemokine levels may support rapid healing and reduced scar formation in the oral mucosal wounds. Another inflammatory pathway that showed tissue specific differences was the JAK-STAT pathway. The JAK-STAT signaling pathway is one of the important pathways to drive biological responses to cytokines [19]. Since there are numbers of cytokines significantly upregulated in the skin wounds, it is not surprising that the JAK-STAT signaling pathway is involved in the inflammatory response to wounding seen in the skin, but not in mucosa (Additional file 3).

Interestingly, the TLRs were differentially expressed in skin and mucosal wounds. TLRs are a group of transmembrane receptors that traditionally are thought only to recognize structurally conserved molecules derived from various organisms including bacteria, viruses, fungi, and parasites [20-22]. When such threats breach physical barriers such as the skin or mucosa, they bind and activate TLRs on immune cells and induce the innate immune response [20,22,23]. However, recent studies strongly suggest that TLRs also recognize endogenous ligands such as heat shock proteins, hyaluronan, fibronectin, and fibrinogen when these molecules are released from damaged tissues or cells resulting from wounding, ischemia, or other injuries independent of the presence of microbes [24,25]. DAVID gene analysis tools indicated that the TLR signaling pathway is involved in healing. TLR2, TLR4, TLR6, and TLR13 are highly expressed and enriched as a significant group in skin wounds, but not in tongue wounds. In a recent study, migration of human epidermal keratinocytes, in *in vitro *scratch wounds, was inhibited by LPS and the inhibition could be blocked partially by anti-TLR4 (75%) and anti-TLR2 (40%) [26]. The emerging roles of TLRs in wound healing has received little experimental attention to date except in chemical or thermal-induced burn injuries [27-31]. For example, TLR4 plays a critical role in microvascular leakage and leukocyte adhesion under the inflammatory conditions associated with nonseptic thermal injury [27]. Severe burn also primes the innate immune system for enhanced TLR2 and TLR4-mediated response which may contribute to the development of heightened systemic inflammation [28]. TLRs have also been demonstrated changes in various skin diseases such as acne, leprosy, psoriasis, and Lyme disease [32]. The importance of TLRs in the injury response is under active investigation by many laboratories.

Beyond the inflammation related elements, several additional pathways and groups of genes that are known to be important to injury responses were differentially expressed in skin and mucosal wounds. The MMPs, a family of zinc-dependent endopeptidases that play a critical role in wound healing process, also showed differential expression in skin and mucosal wounds. MMPs can regulate inflammation, and degrade components of ECM to facilitate the migration of cells. They are involved in tissue remodeling [33-35]. Upregulation of MMP 1a, MMP 1b, MMP 8, MMP 9, MMP 10 and MMP13 seen in early skin wounds, but not in oral mucosal wounds, suggests that cells in the skin reacts to wounding with a much more aggressive production of MMPs than do the cells in mucosa.

Another group of non-inflammatory related genes that were differentially expressed was the peptidases. A number of peptidase genes (Table 8 and additional file 8) that belong to the late upregulated gene cluster were found to only appear in skin wounds. This significantly more robust expression of peptidases in skin may suggest that tissue remodeling or reorganization processes are active in skin wounds at later time points than they are in tongue. This concept is consistent with clinical and pathological observations in these two types of wounds.

On the whole, the differences noted in the transcriptome of oral mucosal and skin wounds are striking, and raise questions as to whether some of the divergence might be due to anatomic variation itself. Oral mucosa and skin are both stratified epithelium, yet these tissues do exhibit multiple microscopic and anatomic differences, such as hair follicles and sweat glands (in skin), and taste buds (in mucosa). Certainly these various differences may account for some of the differential transcriptional events that are observed during the injury response at the two sites. However, studies in palate, buccal mucosa, and tongue models all demonstrate that oral mucosa, regardless of specific anatomical features, heals more rapidly than skin. Thus, the improved response to injury seen in oral mucosa seems to include elements that are not dependent upon adnexal structures.

In the context of our understanding of the wound healing response, it seems likely that many of the observed differences in the transcriptome of skin and mucosal wounds are derived from epithelium rather than connective tissue. An important role for the epithelial response in dictating wound healing outcomes has been suggested by numerous studies [36]. At sites of injury, the epithelium is a rich source of both proinflammatory mediators, such as IL-1, and TNF-α, as well as growth factors, such as vascular endothelial growth factor (VEGF). Keratinocytes are also capable of modulating fibroblast behavior, including collagen synthesis, through the production and release of soluble factors [37,38]. Given the importance of the epithelial response to healing outcomes, at least a portion of the wound healing phenotype probably reflects intrinsic differences in the epithelial response to injury at skin and mucosal sites. In support of this concept, we found that keratinocytes from skin and oral mucosa respond differently to equivalent IL-1β stimulation. Our experiments showed that, following identical stimulation in vitro, skin keratinocytes have an intrinsic capacity to express significantly more IL-6 and TNF-α than mucosal keratinocytes (p < 0.01, Fig. 5). This data strongly supports the concept that keratinocytes from skin and mucosa maintain differential regulatory pathways that lead to differential responsiveness at sites of injury. Such site-specific regulation may be responsible for the increased inflammation that is observed in skin versus mucosal wounds. Additional studies will be needed to isolate the contributions of epithelial and connective tissue responses to injury at the two sites.

## Conclusions

Taken together, the current study provides a unique, comprehensive view of the different gene expression profiles in the process of skin and mucosal wound healing. The results strongly suggests that there is a fundamental difference in intrinsic genetic response to wounding between skin and mucosa, which makes mucosal wounds heal faster and with less inflammation and scar formation. The combination of the current findings with proteomic studies may permit the identification of genes or response elements that are responsible for superior mucosal wound healing and/or the more severe scar formation that is commonly seen in skin wounds.

## Methods

### Animals and wound models

Six- to 8-week-old female Balb/c mice (Harlan Inc., Indianapolis, IN) were anesthetized with ketamine (100 mg/kg) and xylazine (5 mg/kg). For dermal wounds the dorsal skin was shaved, wiped with isopropyl alcohol, and six full-thickness dermal wounds were placed on the opposite sides of the midline using a 1 mm punch biopsy instrument (Acu-Punch, Acuderm Inc., Ft. Lauderdale, FL) [1,2,7]. In a separate group of mice, oral mucosal wounds were placed on the lateral side of the tongue at an equal distance from the midline using the same 1 mm punch biopsy instrument [1,2,7]. At defined intervals (6 hours, 12 hours, 24 hours, day 3, day 5, day 7 and day 10) after injury, wounds and surrounding tissues were removed with a 2 mm biopsy punch and stored in RNAlater (Sigma, St. Louis, MO) until processing. Skin wounds include epidermis, dermis, subcutaneous, and muscle. Tongue wounds involve epithelia, connective tissue and muscle. The time points chosen (6, 12, 24 hours, 3, 5, 7, and 10 days after wounding) covered all these four stages. Six-12 hours, 24 hours-day 3, day 5-7, and day10 correspond to hemostasis, inflammation, proliferation, and remodeling respectively. All 6 skin wounds from each mouse were pooled and used as one sample in the following microarray and RT-PCR analyses. Each time point had 3 mice for either skin or tongue wound group. Additional skin or tongue wounds were made and samples were frozen in TBS tissue freezing medium (Triangle Biomedical Sciences, Durham, NC) for subsequent hematoxylin and eosin (H&E) staining to evaluate the reepithelialization as described in our previous publication [7] (n = 4). All animal procedures were approved by the University of Illinois Institutional Animal Care and Use Committee.

### Total RNA preparation

Total RNA was extracted using TriZol (Invitrogen, Carlsbad, CA) and purified up by RNeasy kit (QIAGEN, Valencia, CA). The integrity (18S/28S) and concentrations of RNA was determined using an Experion (Bio-Rad, Hercules, CA) per the manufacturer's instruction.

### Microarray data collection and data analysis

Total RNA from each sample was labeled and hybridized to Affymetrix GeneChip Mouse Genome 430 v 2.0 chip (Santa Clara, CA) containing 45,037 gene sets according to standard Affymetrix recommended protocols. Each hybridization image was inspected for the following quality metrics: total background, raw noise (Q), average signal present, signal intensity of species-specific house-keeping genes, 3'/5' signal ratio of house-keeping genes, relative signal intensities of labeling controls, absolute signal intensities of hybridization controls, and GCOS scale factors. All 48 hybridizations passed each quality criteria.

Hybridization data was analyzed in 'S-Plus' 6.2 statistical package (Insightful, Palo Alto, CA). Signal intensities were normalized and background was corrected according to Robust Multi-array Average (RMA) method [39]. A one-way ANOVA test was applied to identify significant differentially expressed gene sets across all time points after wounding. Levels of gene expression at each time point in the wounded skin or wounded tongue were compared to normal skin or normal tongue tissue respectively. If any gene was identified as significant differentially expressed at one time point or more after injury, this gene was included for further analyses. Raw p-values were corrected for False Discovery Rate (FDR) according to the Benjamini-Hochberg (BH) procedure [40]. Differentially expressed transcripts were annotated according to Affymetrix's 'NetAffx Analysis Center' (Annotation release R23). A subset of statistically significant transcripts was used for K means clustering in order to analyze patterns of expression changes across all time points. The following filtering criteria were applied: 1) absolute fold change > 2 in at least one time point in relation to normal tissue, and 2) FDR corrected p Value < 1E-5. The microarray data were deposited into the GEO repository and made public on July 20, 2010 with the accession number GSE23006.

### Skin and mucosal keratinocyte isolation and cell culture

Human skin and oral mucosal (palate) tissues were obtained from healthy adult donors after consent under a protocol approved by Institutional Review Board at the University of Illinois at Chicago. The procedures for isolating keratinocyte from skin and palate tissues were the same as described previously [1]. Keratinocytes from both skin and mucosa were plated in 12-well plates at a density of 4.5 × 10^4 ^cells/well in KBM-2 medium (Lonza, Basel, Switzerland) and incubated overnight. The following day, the cells were treated with human recombinant IL-1β (50 ng/ml) (PeproTech, Rocky Hills, NJ) or medium only for 24 hours. The cells were then harvested using Trizol followed by total RNA isolation and DNase I (Invitrogen) treatment. Three independent isolates of primary keratinocytes were obtained from normal human skin and oral mucosa. One isolate from skin and one isolate from mucosa were from the same individual and were prepared in our lab. Two additional cultures of primary mucosal keratinocytes were also isolated in our lab. The two additional isolates of skin keratinocyte were purchased from Lonza. Basel, Switzerland. Similar results were obtained from the skin keratinocytes isolated in our lab and purchased from Lonza. Representative pictures of skin and mucosal keratinocytes isolated in our lab are shown in (Additional file 11). From the morphology view, there were no contaminations from other cells.

### Real-Time RT-PCR Verification of Affymetrix GeneChip Data

In order to verify the results obtained from the microarray analysis, we chose 7 genes including CCL2, CCL27, CXCL2, CSF1, IL-1β, collagen Iα2, and collagen III α1, which showed different patterns of differential expression. These genes were subjected to validation by real-time PCR. Three time points including normal tissue, 12 h, and day 10 wounds were used for the analysis of Collagen Ia2 and Collagen III a1 by real-time RT-PCR; normal tissue, 24 h, and day 10 wounds were used for the rest of the genes. At least three primers pairs were designed for each transcript. Sequence regions used for Affymetrix gene sets design were targeted for real-time validation as well. The online application PrimerQuest (IDT, Coralville, IA) was used for primer design. Amplification specificity was tested by RT-PCR with cDNA templates and mouse genomic DNA. The best primer pairs were selected for use in real-time RT-PCR reactions (Table 10). All primers pairs with the exception of IL-1β were located across exon/intron boundaries and produced different size PCR products from genomic DNA and cDNA templates. For IL-1β primers spanning exon/intron boundaries could not be identified. For the IL-1β analysis, RNA was DNAse I treated prior to real-time RT-PCR validation. One-step real-time RT-PCR was preformed as described for QuantiTect SYBR Green RT-PCR kit (QIAGEN, Valencia, CA). The PCR amplification parameters recommended by the manufacturer were used. Three biological replications (each in three technical replications) were performed for each group of samples. Glucuronidase β (Gusb) and β-actin were selected as normalization controls.

**Table 10:** Primer sets for real time RT-PCR

Genes	Forward primers (5'--3')	Reverse primers (5'--3')
COLIa2	AGGCGTGAAAGGACACAGTGGTAT	TCCTGCTTGACCTGGAGTTCCATT
COLIIIa1	AGCTTTGTGCAAAGTGGAACCTGG	CAAGGTGGCTGCATCCCAATTCAT
CXCL2	AAAGTTTGCCTTGACCCTGAAGCC	TCCAGGTCAGTTAGCCTTGCCTTT
CCL2	AGCAGGTGTCCCAAAGAAGCTGTA	AAAGGTGCTGAAGACCTTAGGGCA
CCL27	CTGGCATCCGTGGAACAAGACTAA	CTGCAGTTCCATGTGGACAATCCT
CSF1	ATCCTAGTCTTGCTGACTGTTGGG	ATCCAATGTCTGAGGGTCTCGATG
IL-1β	TGAAGAAGAGCCCATCCTCTGTGA	TGTCTAATGGGAACGTCACACACC
IL-6	CACCGGGAACGAAAGAGAAG	CCCAGGGAGAAGGCAACTG
TNF-α	TCTTCTCGAACCCCGAGTGA	CCTCTGATGGCACCACCAG
GAPDH	CAGGGCTGCTTTTAACTCTGG	TGGGTGGAATCATATTGGAACA

Real time PCR was also used to determine the mRNA expression of IL-6 and TNF-α by skin and mucosal keratinocytes after being treated by IL-1β. 2^-∆∆Ct ^method was used to calculate fold changes of IL-6 and TNF-α mRNA expression after IL-1β treatment compared to medium treated cells. Student *t *test was used to determine the statistical difference between the fold changes in skin and mucosa. A p value less than 0.05 was considered statistically significant.

## Authors' contributions

LC and ZHA carried out microarray and statistical analyses, LC and SG carried out keratinocyte isolation and cell culture, LC, ZHA, PTM, TAM, and LAD participated in the design of the study, LC, ZHA and LAD drafted the manuscript. All authors read and approved the final manuscript.

## Supplementary Material

Additional file 1 **Cytokine-cytokine receptor interaction pathway in early upregulated genes (clusters 1, 2, and 3)**. Click here for file

Additional file 2 **Toll-like receptor signaling pathway in skin early upregulated genes (clusters 1, 2, and 3)**. Click here for file

Additional file 3 **JAK-STAT signaling pathway in skin early upregulated genes (clusters 1, 2, and 3)**. Click here for file

Additional file 4 **Early upregulated skin clusters 1, 2, and 3 functional classification**. Click here for file

Additional file 5 **Early upregulated tongue cluster 1, 2, and 3 functional classification**. Click here for file

Additional file 6 **Early downregulated skin cluster 4 functional classification**. Click here for file

Additional file 7 **Early downregulated tongue cluster 4 functional classification**. Click here for file

Additional file 8 **Late upregulated skin cluster 5 functional classification**. Click here for file

Additional file 9 **Late upregulated tongue cluster 5 functional classification**. Click here for file

Additional file 10 **Baseline levels of cytokines/chemokines in skin and tongue**. Click here for file

Additional file 11 **Oral and skin keratinocytes isolated from adult human palate and skin**. Click here for file
