# Supplementary material for: Positional differences in the wound transcriptome of skin and oral mucosa
Source: BMC Genomics. 2010 Aug 12;11:471. doi: 10.1186/1471-2164-11-471 (PMC3091667; doi:10.1186/1471-2164-11-471)

Additional file 2. Toll-like receptor signaling pathway in skin early upregulated genes (clusters 1, 2, and 3)

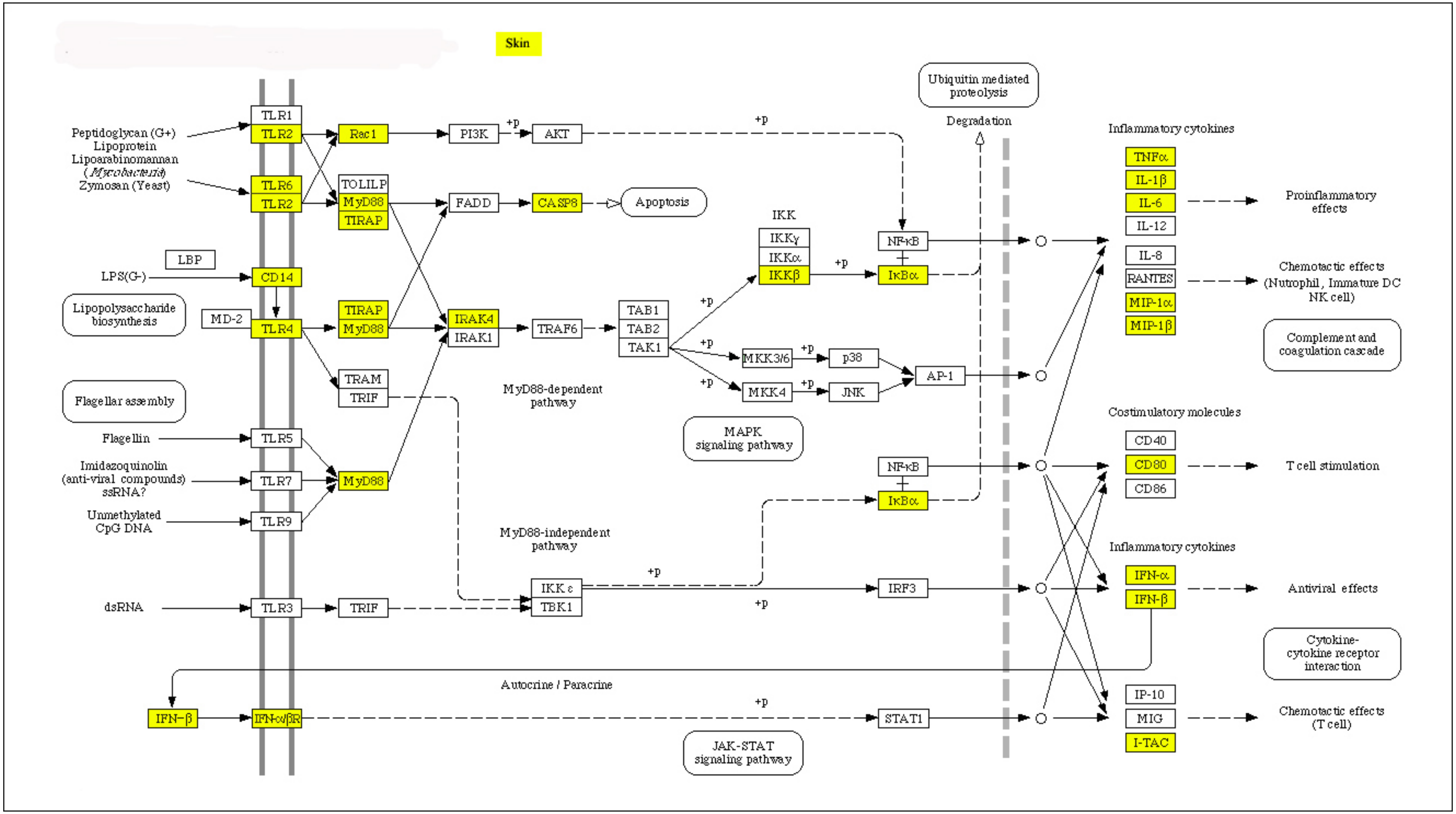

Supplement: Additional file 2 — Toll-like receptor signaling pathway in skin early upregulated genes (clusters 1, 2, and 3). [file 1471-2164-11-471-S2.PDF]
