# Supplementary material for: Positional differences in the wound transcriptome of skin and oral mucosa
Source: BMC Genomics. 2010 Aug 12;11:471. doi: 10.1186/1471-2164-11-471 (PMC3091667; doi:10.1186/1471-2164-11-471)
Supplement: Additional file 4 — Early upregulated skin clusters 1, 2, and 3 functional classification. [file 1471-2164-11-471-S4.PDF]

Additional file 4. Early upregulated skin clusters 1, 2, and 3 functional classification

**Functional Group 1 (Probe set IDs)**	**Interferon α, β and δ/cytokine, Enrichment Score: 9.77**
1422403_at	INTERFERON ALPHA FAMILY, GENE 5
1419529_at	INTERLEUKIN 23, ALPHA SUBUNIT P19
1422305_at	INTERFERON BETA 1, FIBROBLAST
1422403_at	INTERFERON ALPHA FAMILY, GENE 1
1426181_a_at	INTERLEUKIN 24
1422403_at	INTERFERON ALPHA FAMILY, GENE 7
1422403_at	INTERFERON ALPHA FAMILY, GENE 2
1422403_at	INTERFERON ALPHA FAMILY, GENE 6
1422403_at	INTERFERON ALPHA FAMILY, GENE 9
1419427_at	COLONY STIMULATING FACTOR 3 (GRANULOCYTE)
1422403_at	INTERFERON ALPHA FAMILY, GENE B
1422403_at	ALPHA-INTERFERON
1422403_at	INTERFERON ALPHA 8/6 PRECURSOR; IFNA8/6
1422403_at	INTERFERON ALPHA FAMILY, GENE 12
1422403_at	INTERFERON ALPHA 6T
1422403_at	INTERFERON ALPHA 14
**Functional Group 2**	**Chemokines, Enrichment Score: 9.58**
1421578_at	CHEMOKINE (C-C MOTIF) LIGAND 4
1421228_at	CHEMOKINE (C-C MOTIF) LIGAND 7
1420380_at	CHEMOKINE (C-C MOTIF) LIGAND 2
1419561_at	CHEMOKINE (C-C MOTIF) LIGAND 3
1422029_at	CHEMOKINE (C-C MOTIF) LIGAND 20
1449984_at	CHEMOKINE (C-X-C MOTIF) LIGAND 2
1419209_at, 1441855_x_at, 1457644_s_at	CHEMOKINE (C-X-C MOTIF) LIGAND 1
1438148_at	GENE MODEL 1960, (NCBI)
1448859_at	CHEMOKINE (C-X-C MOTIF) LIGAND 13
1419728_at	CHEMOKINE (C-X-C MOTIF) LIGAND 5
1418480_at	CHEMOKINE (C-X-C MOTIF) LIGAND 7
1419697_at	CHEMOKINE (C-X-C MOTIF) LIGAND 11
**Functional Group 3**	**Cell differentiation/apoptosis, Enrichment Score: 3.85**
1416881_at	MYELOID CELL LEUKEMIA SEQUENCE 1
1418835_at	PLECKSTRIN HOMOLOGY-LIKE DOMAIN, FAMILY A, MEMBER 1
1422308_a_at	LECTIN, GALACTOSE BINDING, SOLUBLE 7
1418649_at	EGL NINE HOMOLOG 3 (C. ELEGANS)
1419004_s_at	B-CELL LEUKEMIA/LYMPHOMA 2 RELATED PROTEIN A1A
1419004_s_at	B-CELL LEUKEMIA/LYMPHOMA 2 RELATED PROTEIN A1D
1419004_s_at	B-CELL LEUKEMIA/LYMPHOMA 2 RELATED PROTEIN A1B
1434350_at	AXIN1 UP-REGULATED 1
**Functional Group 4**	**Positive regulation of protein kinase activity, Enrichment Score: 3.68**
1437676_at	SPERM ASSOCIATED ANTIGEN 9

1418685_at	TOLL-INTERLEUKIN 1 RECEPTOR (TIR) DOMAIN-CONTAINING ADAPTOR PROTEIN
1449773_s_at, 1450971_at	GROWTH ARREST AND DNA-DAMAGE-INDUCIBLE 45 BETA
1423122_at	ARGININE VASOPRESSIN-INDUCED 1
**Functional Group 5**	**Regulation of metabolic/biological process, Enrichment Score: 2.91**
1418842_at	HEMATOPOIETIC CELL SPECIFIC LYN SUBSTRATE 1
1437270_a_at, 1437271_at	CARDIOTROPHIN-LIKE CYTOKINE FACTOR 1
1432826_a_at, 1457952_at	CD80 ANTIGEN
1451318_a_at	YAMAGUCHI SARCOMA VIRAL (V-YES-1) ONCOGENE HOMOLOG
**Functional Group 6**	**Pattern recognition receptors (TLR related), Enrichment Score: 2.82**
1420699_at	C-TYPE LECTIN DOMAIN FAMILY 7, MEMBER A
1419132_at	TOLL-LIKE RECEPTOR 2
1419272_at	MYELOID DIFFERENTIATION PRIMARY RESPONSE GENE 88
1449399_a_at	INTERLEUKIN 1 BETA
1418162_at, 1418163_at, 1430695_at, 1442827_at	TOLL-LIKE RECEPTOR 4
1421352_at	TOLL-LIKE RECEPTOR 6
1457753_at	TOLL-LIKE RECEPTOR 13
**Functional Group 7**	**Ribosome/organelle organization, Enrichment Score: 2.5**
1428315_at	EBNA1 BINDING PROTEIN 2
1422767_at, 1450742_at	BYSTIN-LIKE
1433502_s_at	TSR1, 20S RRNA ACCUMULATION, HOMOLOG (YEAST)
1452414_s_at	RIKEN CDNA 4933411H20 GENE
1452454_at	SDA1 DOMAIN CONTAINING 1
1448480_at	NUCLEAR IMPORT 7 HOMOLOG (S. CEREVISIAE)
1454841_at	RIKEN CDNA 5730405K23 GENE
1423823_at	RIKEN CDNA 2610012O22 GENE
**Functional Group 8**	**Matrix metallopeptidase, enrichment Score: 2.04**
1416298_at, 1448291_at	MATRIX METALLOPEPTIDASE 9
1417256_at	MATRIX METALLOPEPTIDASE 13
1420450_at	MATRIX METALLOPEPTIDASE 10
1422175_at	MATRIX METALLOPEPTIDASE 1A (INTERSTITIAL COLLAGENASE)
1422273_at	MATRIX METALLOPEPTIDASE 1B (INTERSTITIAL COLLAGENASE)
1449366_at	MATRIX METALLOPEPTIDASE 8
