# Supplementary material for: Positional differences in the wound transcriptome of skin and oral mucosa
Source: BMC Genomics. 2010 Aug 12;11:471. doi: 10.1186/1471-2164-11-471 (PMC3091667; doi:10.1186/1471-2164-11-471)
Supplement: Additional file 5 — Early upregulated tongue cluster 1, 2, and 3 functional classification. [file 1471-2164-11-471-S5.PDF]

**Additional file 5. Early upregulated tongue cluster 1, 2, and 3 functional classification**

| Functional Group 1 (Probe set IDs) | Chemokines,  enrichment Score: 6.46 |
|---|---|
| 1421578_at | CHEMOKINE (C-C MOTIF) LIGAND 4 |
| 1419282_at | CHEMOKINE (C-C MOTIF) LIGAND 12 |
| 1421228_at | CHEMOKINE (C-C MOTIF) LIGAND 7 |
| 1418126_at | CHEMOKINE (C-C MOTIF) LIGAND 5 |
| 1420380_at | CHEMOKINE (C-C MOTIF) LIGAND 2 |
| 1449984_at | CHEMOKINE (C-X-C MOTIF) LIGAND 2 |
| 1419209_at, 1457644_s_at | CHEMOKINE (C-X-C MOTIF) LIGAND 1 |
| 1419728_at | CHEMOKINE (C-X-C MOTIF) LIGAND 5 |
| 1418930_at | CHEMOKINE (C-X-C MOTIF) LIGAND 10 |
| 1419697_at | CHEMOKINE (C-X-C MOTIF) LIGAND 11 |
| **Functional Group 2** | **Negative regulation of cell proliferation, Enrichment Score: 3.34** |
| 1423754_at | INTERFERON INDUCED TRANSMEMBRANE PROTEIN 3 |
| 1418612_at | SCHLAFEN 1 |
| 1450322_s_at | SCHLAFEN 3 |
| 1450165_at | SCHLAFEN 2 |
| **Functional Group 3** | **GTP binding, Enrichment Score: 3.08** |
| 1435652_a_at | GUANINE NUCLEOTIDE BINDING PROTEIN, ALPHA INHIBITING 2 |
| 1459315_at | RAB5C, MEMBER RAS ONCOGENE FAMILY |
| 1419042_at, 1419043_a_at | INTERFERON INDUCIBLE GTPASE 1 |
| 1420549_at | GUANYLATE NUCLEOTIDE BINDING PROTEIN 1 |
| 1418240_at, 1435906_x_at | GUANYLATE NUCLEOTIDE BINDING PROTEIN 2 |
| 1423619_at | RAS, DEXAMETHASONE-INDUCED 1 |
| 1451905_a_at | MYXOVIRUS (INFLUENZA VIRUS) RESISTANCE 1 |
| 1419676_at | MYXOVIRUS (INFLUENZA VIRUS) RESISTANCE 2 |
| 1418392_a_at | GUANYLATE NUCLEOTIDE BINDING PROTEIN 4 |
| 1417793_at | INTERFERON INDUCIBLE GTPASE 2 |
| 1449009_at | T-CELL SPECIFIC GTPASE |
| 1422562_at | RAS-RELATED ASSOCIATED WITH DIABETES |
| 1417141_at, 1458589_at | INTERFERON GAMMA INDUCED GTPASE |
| 1425156_at, 1434380_at | RIKEN CDNA 9830147J24 GENE |
| **Functional Group 4** | **Keratinization/epidermal development, Enrichment Score: 2.27** |
| 1422240_s_at | SMALL PROLINE-RICH PROTEIN 2H |
| 1422963_at | SMALL PROLINE-RICH PROTEIN 2I |
| 1449833_at | SMALL PROLINE-RICH PROTEIN 2F |
| 1420771_at | SMALL PROLINE-RICH PROTEIN 2D |
| 1450811_at | SMALL PROLINE-RICH PROTEIN 2J |
