# Supplementary material for: Positional differences in the wound transcriptome of skin and oral mucosa
Source: BMC Genomics. 2010 Aug 12;11:471. doi: 10.1186/1471-2164-11-471 (PMC3091667; doi:10.1186/1471-2164-11-471)
Supplement: Additional file 6 — Early downregulated skin cluster 4 functional classification. [file 1471-2164-11-471-S6.PDF]

Additional file 6. Early downregulated skin cluster 4 functional classification

Functional Group 1 (Probe set IDs)	Transcription regulation/DNA binding, Enrichment Score: 3.0
1435561_at	ETS2 REPRESSOR FACTOR
1419155_a_at, 1419156_at, 1433575_at	SRY-BOX CONTAINING GENE 4
1416155_at	HIGH MOBILITY GROUP BOX 3
1425048_a_at, 1435324_x_at, 1439463_x_at	HIGH MOBILITY GROUP BOX 1
1439136_at	SINGLE-STRANDED DNA BINDING PROTEIN 3
1449058_at	GLI-KRUPPEL FAMILY MEMBER GLI1
1446086_s_at, 1459211_at	GLI-KRUPPEL FAMILY MEMBER GLI2
1425099_a_at	ARYL HYDROCARBON RECEPTOR NUCLEAR TRANSLOCATOR-LIKE
1429428_at	TRANSCRIPTION FACTOR 7-LIKE 2, T-CELL SPECIFIC, HMG-BOX
1444043_at, 1456067_at	GLI-KRUPPEL FAMILY MEMBER GLI3
1448601_s_at	HOMEO BOX, MSH-LIKE 1
1457480_at	POLYCOMB GROUP RING FINGER 3
1433632_at	INTERFERON REGULATORY FACTOR 2 BINDING PROTEIN 2
1448674_at	RING FINGER PROTEIN 25
1428466_at	CHROMODOMAIN HELICASE DNA BINDING PROTEIN 3
1448361_at	TETRATRICOPEPTIDE REPEAT DOMAIN 3
1434017_at	ZINC AND RING FINGER 2
1435340_at	JUMONJI DOMAIN CONTAINING 2A
1450090_at	ZINC FINGER PROTEIN 101
1450151_at	ZINC FINGER PROTEIN 316
1434311_at	CCR4-NOT TRANSCRIPTION COMPLEX, SUBUNIT 6-LIKE
1431099_at	HOMEO BOX D8
1450117_at	TRANSCRIPTION FACTOR 3
1452856_at	HCF-BINDING TRANSCRIPTION FACTOR ZHANGFEI
1429739_a_at	ZINC FINGER PROTEIN 278
1438072_at, 1448288_at	NUCLEAR FACTOR I/B
1454675_at	THYROID HORMONE RECEPTOR ALPHA
1460577_at	JUNCTION-MEDIATING AND REGULATORY PROTEIN
1426208_x_at	PLEIOMORPHIC ADENOMA GENE-LIKE 1
1451285_at, 1451286_s_at	FUSION, DERIVED FROM T(12;16) MALIGNANT LIPOSARCOMA (HUMAN)
1418317_at	LIM HOMEOBOX PROTEIN 2
1422032_a_at	ZINC FINGER, A20 DOMAIN CONTAINING 3
1418408_at	ZINC FINGER, AN1-TYPE DOMAIN 1
1450760_a_at	INHIBITOR OF GROWTH FAMILY, MEMBER 3
1446929_at	BTB AND CNC HOMOLOGY 2
1441140_at	ARGININE GLUTAMIC ACID DIPEPTIDE (RE) REPEATS
1418894_s_at, 1449261_at	PRE B-CELL LEUKEMIA TRANSCRIPTION FACTOR 2
1425628_a_at	GENERAL TRANSCRIPTION FACTOR II I

1445873_at	TRANSCRIPTION FACTOR DP 2
1436217_at	ZINC FINGER PROTEIN 148
1434002_at	CHECKPOINT SUPPRESSOR 1
1450194_a_at	MYELOBLASTOSIS ONCOGENE
1428469_a_at, 1452792_at	DAZ INTERACTING PROTEIN 1
1424407_s_at	DNA SEGMENT, CHR 15, BRIGHAM & WOMEN'S GENETICS 0580 EXPRESSED
1416638_at	SAL-LIKE 2 (DROSOPHILA)
1429086_at	RIKEN CDNA 0610015A08 GENE
1438861_at	BASONUCLIN 2
1436952_at	KRUPPEL-LIKE FACTOR 9
1416868_at	CYCLIN-DEPENDENT KINASE INHIBITOR 2C (P18, INHIBITS CDK4)
1435282_at	GENE MODEL 967, (NCBI)
1455547_at	ZINC FINGER CCCH-TYPE CONTAINING 7B
1437291_at	RIKEN CDNA 2700081O15 GENE
