# Supplementary material for: Positional differences in the wound transcriptome of skin and oral mucosa
Source: BMC Genomics. 2010 Aug 12;11:471. doi: 10.1186/1471-2164-11-471 (PMC3091667; doi:10.1186/1471-2164-11-471)
Supplement: Additional file 7 — Early downregulated tongue cluster 4 functional classification. [file 1471-2164-11-471-S7.PDF]

**Additional file 7. Early downregulated tongue cluster 4 functional classification**

| Functional Group 1 (Probe set IDs) | Transcription regulation/DNA binding, Enrichment Score: 1.25 |
|---|---|
| 1451285_at | FUSION, DERIVED FROM T(12;16) MALIGNANT LIPOSARCOMA (HUMAN) |
| 1450760_a_at | INHIBITOR OF GROWTH FAMILY, MEMBER 3 |
| 1424407_s_at | DNA SEGMENT, CHR 15, BRIGHAM & WOMEN'S GENETICS 0580 EXPRESSED |
| 1418894_s_at | PRE B-CELL LEUKEMIA TRANSCRIPTION FACTOR 2 |
| 1426464_at | NUCLEAR RECEPTOR SUBFAMILY 1, GROUP D, MEMBER 1 |
