# Supplementary material for: Positional differences in the wound transcriptome of skin and oral mucosa
Source: BMC Genomics. 2010 Aug 12;11:471. doi: 10.1186/1471-2164-11-471 (PMC3091667; doi:10.1186/1471-2164-11-471)
Supplement: Additional file 8 — Late upregulated skin cluster 5 functional classification. [file 1471-2164-11-471-S8.PDF]

Additional file 8. Late upregulated skin cluster 5 functional classification

**Functional Group 1 (Probe set IDs)**	**Collagen, Enrichment Score: 9.61**
1427884_at	PROCOLLAGEN, TYPE III, ALPHA 1
1418599_at, 1449154_at	PROCOLLAGEN, TYPE XI, ALPHA 1
1429549_at	PROCOLLAGEN, TYPE XXVII, ALPHA 1
1416740_at, 1416741_at	PROCOLLAGEN, TYPE V, ALPHA 1
1448590_at	PROCOLLAGEN, TYPE VI, ALPHA 1
1416414_at	ELASTIN MICROFIBRIL INTERFACER 1
1422606_at	C1Q AND TUMOR NECROSIS FACTOR RELATED PROTEIN 3
1426947_x_at, 1452250_a_at	PROCOLLAGEN, TYPE VI, ALPHA 2
1423110_at, 1446326_at	PROCOLLAGEN, TYPE I, ALPHA 2
1418440_at, 1447819_x_at, 1455627_at	PROCOLLAGEN, TYPE VIII, ALPHA 1
1452968_at	COLLAGEN TRIPLE HELIX REPEAT CONTAINING 1
1427168_a_at, 1428455_at	PROCOLLAGEN, TYPE XIV, ALPHA 1
1450625_at	PROCOLLAGEN, TYPE V, ALPHA 2
**Functional Group 2**	**Serine-type endopeptidase activity, Enrichment Score: 3.05**
1419722_at	PROTEASE, SERINE, 19 (NEUROPSIN)
1426175_a_at	TRYPTASE ALPHA/BETA 1
1455848_at	TRANSMEMBRANE PROTEASE, SERINE 11A
1434195_at	PROTEASE, SERINE, 35
**Functional Group 3**	**EGF-like calcium-binding, Enrichment Score: 2.83**
1460574_at	HYPOTHETICAL PROTEIN 9430004M15
1417439_at	CD248 ANTIGEN, ENDOSIALIN
1446048_at, 1450757_at	CADHERIN 11
1449388_at	THROMBOSPONDIN 4
**Functional Group 4**	**Metalloendopeptidase activity, Enrichment Score: 2.22**
1417282_at	MATRIX METALLOPEPTIDASE 23
1421171_at, 1421172_at	A DISINTEGRIN AND METALLOPEPTIDASE DOMAIN 12 (MELTRIN ALPHA)
1439827_at	EXPRESSED SEQUENCE AI605170
1439604_at	A DISINTEGRIN-LIKE AND METALLOPETIDASE (REPROLYSIN TYPE) WITH THROMBOSPONDIN TYPE 1 MOTIF, 16
**Functional Group 5**	**Cytoskeleton, Enrichment Score: 2.08**
1415927_at	ACTIN, ALPHA, CARDIAC
1418726_a_at, 1424967_x_at	TROPONIN T2, CARDIAC
1427115_at	MYOSIN, HEAVY POLYPEPTIDE 3, SKELETAL MUSCLE, EMBRYONIC
1426650_at	MYOSIN, HEAVY POLYPEPTIDE 13, SKELETAL MUSCLE
1450813_a_at	TROPONIN I, SKELETAL, SLOW 1
1449207_a_at	KINESIN FAMILY MEMBER 20A
1418370_at	TROPONIN C, CARDIAC/SLOW SKELETAL
1440990_at	CDNA SEQUENCE BC056349
