# Supplementary material for: Positional differences in the wound transcriptome of skin and oral mucosa
Source: BMC Genomics. 2010 Aug 12;11:471. doi: 10.1186/1471-2164-11-471 (PMC3091667; doi:10.1186/1471-2164-11-471)
Supplement: Additional file 9 — Late upregulated tongue cluster 5 functional classification. [file 1471-2164-11-471-S9.PDF]

Additional file 9. Late upregulated tongue cluster 5 functional classification

**Functional Group 1 (Probe set IDs)**	**Collagen, Enrichment Score: 6.58**
1416741_at, 1416740_at	PROCOLLAGEN, TYPE V, ALPHA 1
1423669_at	PROCOLLAGEN, TYPE I, ALPHA 1
1427884_at	PROCOLLAGEN, TYPE III, ALPHA 1
1423110_at	PROCOLLAGEN, TYPE I, ALPHA 2
1422437_at, 1450625_at	PROCOLLAGEN, TYPE V, ALPHA 2
1452250_a_at	PROCOLLAGEN, TYPE VI, ALPHA 2
1422606_at	C1Q AND TUMOR NECROSIS FACTOR RELATED PROTEIN 3
1452968_at	COLLAGEN TRIPLE HELIX REPEAT CONTAINING 1
1427168_a_at	PROCOLLAGEN, TYPE XIV, ALPHA 1
**Functional Group 2**	**Cytoskeleton, Enrichment Score: 3.57**
1418370_at	TROPONIN C, CARDIAC/SLOW SKELETAL
1415927_at	ACTIN, ALPHA, CARDIAC
1427115_at	MYOSIN, HEAVY POLYPEPTIDE 3, SKELETAL MUSCLE, EMBRYONIC
1418726_a_at, 1424967_x_at	TROPONIN T2, CARDIAC
1450813_a_at	TROPONIN I, SKELETAL, SLOW 1
