# Supplementary material for: Positional differences in the wound transcriptome of skin and oral mucosa
Source: BMC Genomics. 2010 Aug 12;11:471. doi: 10.1186/1471-2164-11-471 (PMC3091667; doi:10.1186/1471-2164-11-471)
Supplement: Additional file 10 — Baseline levels of cytokines/chemokines in skin and tongue. [file 1471-2164-11-471-S10.PDF]

Additional file 10. Baseline levels of cytokines/ chemokines in skin and tongue*

	Normal skin	Normal tongue
IFN-α	6.3	6.0
IFN-β	5.2	4.9
IL-23	4.6	4.2
IL-24	4.3	4.4
CSF-3	5.5	5.3
CCL3	4.3	4.3
CCL20	6.8	5.4
CXCL3	3.0	2.6
CXCL7	4.3	4.5
CXCL13	7.7	8.7

*Baseline (log2 expression) levels are shown for ten mediators that were found to be upregulated in skin wounds only.
