# Supplementary material for: Positional differences in the wound transcriptome of skin and oral mucosa
Source: BMC Genomics. 2010 Aug 12;11:471. doi: 10.1186/1471-2164-11-471 (PMC3091667; doi:10.1186/1471-2164-11-471)
Supplement: Additional file 11 — Oral and skin keratinocytes isolated from adult human palate and skin. [file 1471-2164-11-471-S11.PDF]

Additional file 11. Oral and skin keratinocytes isolated from adult human palate and skin

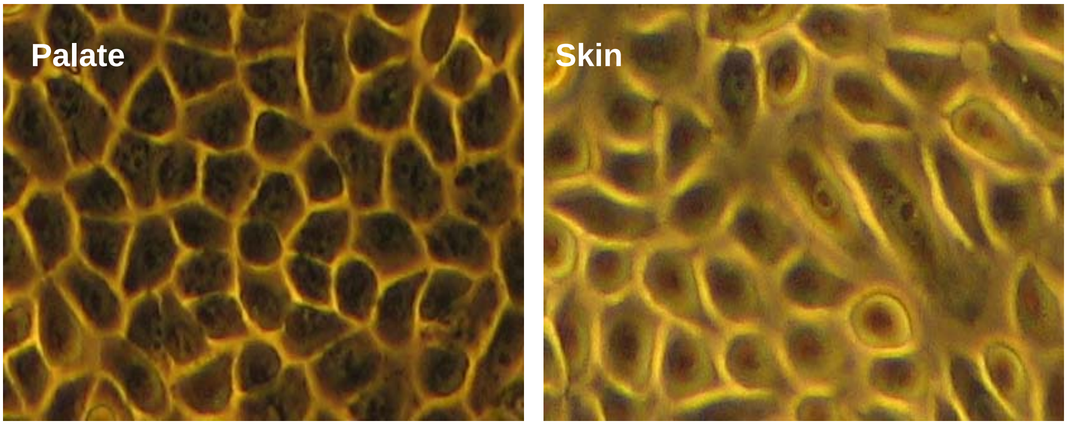

The morphology of primary isolates of keratinocytes from healthy adult human palate and skin is shown. Isolates were prepared as described in the Materials and Methods.
